# A Perspective on the Potential of Human iPS Cell-Based Therapies for Muscular Dystrophies: Advancements so far and Hurdles to Overcome

**DOI:** 10.4172/2157-7633.1000e113

**Published:** 2013-05-02

**Authors:** Radbod Darabi, Rita C.R. Perlingeiro

**Affiliations:** Lillehei Heart Institute, Department of Medicine, University of Minnesota, 312 Church Street SE, Minneapolis, MN 55455, USA

Pluripotent stem cells [1,2] are well-known for their unique self-renewal and differentiation capabilities, which make these cells very favorable for cell-based therapeutic applications in degenerative disorders such as muscular dystrophies. Major concerns associated with embryonic stem (ES) cells, such as immunological compatibility and ethical considerations had limited their clinical applications [3]. However, the technology of reprogramming somatic cells into induced pluripotent stem (iPS) cells provides a new breath of hope for a potential therapeutic application in incurable diseases [4–6]. iPS cells have been generated initially from mouse and later from human somatic cells by introduction of the four reprogramming transcription factors using retroviral vectors. More recently, this has been accomplished by using non-integrating vectors [7,8], which is much safer as the associated risk of insertional mutagenesis is significantly reduced. Consequently, by proper differentiation of iPS cells into tissue specific progenitors, one can foresee the therapeutic application of iPS cells in a relatively near future, upon extensive testing for the safety of these cell preparations is achieved.

Muscular dystrophies (MDs) are an heterogeneous group of degenerative disorders caused by various gene defects that lead to progressive muscle damage, defective regeneration and fibrosis [9]. Among these, Duchene Muscular Dystrophy (DMD) is the most common type, and is caused by mutations in the dystrophin gene [10]. DMD is characterized by progressive muscle weakness and atrophy, leading patients to be confined to a wheelchair before their teenage years and eventual death due to respiratory and/or cardiac failure. To date, there is no definitive treatment for DMD or any other type of MD. Reprogramming technology potentially allows for a cell replacement therapy using patient-derived iPS cells, which combined with gene correction, and subsequent controlled differentiation into myogenic progenitors, could be used clinically in the autologous transplantation setting. In the current perspective, we review recent studies in this field, and point out the advantages and the shortcomings of each approach.

## Gene Correction of Human DMD iPS Cells using a Human Artificial Chromosome (HAC)

This is the first report describing genetic correction in human DMD iPS cells [11]. The authors utilized a HAC containing the complete human dystrophin sequence (DYS-HAC). The use of HAC for gene therapy has certain advantages including the ability to carry large inserts such as the dystrophin gene, stable episomal maintenance, and importantly, minimum risk of insertional mutagenesis. The DMD mutation corrected in this study was a large deletion of axons 4-43, a mutation not suitable for conventional gene correction methods. The DYS-HAC was transferred via microcell-mediated chromosome transfer (MMCT) into patient fibroblasts and then corrected fibroblasts were used to generate iPS cells. Using FISH analysis, the authors showed stable maintenance of the DYS-HAC in long-term cultures of corrected iPS cells. In teratoma assays, they detected the presence of DYS-HAC in 90% of the cells as well as dystrophin expression in muscle-like tissues within the tumor. For potential clinical application, it will be necessary for corrected DMD cells to be differentiated *in vitro* into myogenic progenitors, and used for *in vivo* regeneration in an animal model for DMD.

## Derivation of Myogenic Progenitors from Human iPS Cells using Pax7

In this report from our group [12], we have applied conditional expression of Pax7, a critical molecular regulator of the skeletal myogenic program, to efficiently promote myogenesis from human ES/iPS cells, a method that worked well in the mouse system [13–15]. A tet-on inducible Pax7 lentiviral vector was introduced into the iPS cells and upon differentiation of these cells into early mesodermal progenitors, Pax7 was induced by adding doxycycline to the cultures, which promoted the generation of proliferating myogenic precursors. Following FACS purification, Pax7^+^ (GFP^+^) cells expanded exponentially *in vitro*, and expressed CD56, M-cadherin and α7 integrin, surface markers associated with early myogenic cells. Following transplantation into an immunodeficient mouse model of DMD, human iPS-derived skeletal myogenic progenitors engrafted into diseased muscle, restored dystrophin expression, improved contractility and seeded the satellite cell compartment. Moreover, long-term engraftment was detected in recipient mice 46 weeks post-transplant. By using non-integrating methods for Pax7 induction, in combination with gene correction approaches, such as DYS-HAC or nuclease mediated gene correction strategies [16–19], one can envision a potential strategy for gene/cell-based therapy in MDs.

## Differentiation of Human iPS Cells into Myogenic Cells using MyoD

Recently, two research groups have used MyoD for myogenic induction of differentiating human iPS cells. In the first study, Tedesco and colleagues [20] have described a novel approach to differentiate human iPS cells into mesoangioblast-like cells, a cell population they have demonstrated to have skeletal muscle regeneration potential [21,22]. Due to the limitation in obtaining sufficient numbers of mesoangioblasts from the vessels of patient biopsies, a protocol to generate these cells from patient-specific iPS was sought. In this investigation, the authors succeeded in doing so using iPS cells from Limb-Girdle MD type 2D (LGMD2D) patients, which are deficient in α-sarcoglycan. A tamoxifen inducible MyoD lentiviral vector was used to promote the *in vitro* myogenic differentiation of these cells into mature myotubes. Using a lentiviral vector encoding human α-sarcoglycan under a muscle specific promoter, corrected human iPS cell-derived mesoangioblast-like cells were shown to engraft and express the missing protein in a mouse model of LGMD2D (Sgca-null/scid/beige) following intramuscular and intra-arterial delivery. One great advantage of this method is the ability of engraftment following systemic delivery.

In the second study, Gougenege and colleagues [23] utilized a two-step protocol, in which human ES and DMD iPS cells were initially differentiated into CD73^+^ mesenchymal-like cells, and then infected with an adenoviral vector expressing MyoD under a ubiquitous promoter (CAG) to generate myogenic cells. Following transplantation into damaged muscles of an immunodeficient mouse model of DMD (*Rag/mdx*), iPS-derived myogenic cells engrafted and differentiated into mature myofibers expressing human spectrin. Although myogenic potential of human ES-cell derived CD73^+^ mesenchymal cells had been reported previously by Barberi and colleagues [24], this approach has not been successfully replicated by other laboratories. By tweaking the protocol and by over-expressing MyoD, these authors have improved differentiation efficiency and reduced culture duration. Another advantage of this study is the use of non-integrating adenoviruses for MyoD induction, reducing the risk of mutagenesis.

A possible limitation associated with the use of MyoD is its direct effect to induce cell cycle arrest [25], which may limit myogenic cell expansion to the levels needed for cell therapy as well as their *in vivo* self-renewal. Recently, Iacovino and colleagues [26] have used Myf5 to induce myogenesis in human ES cells. By inserting the myogenic regulator gene Myf5 into an inducible cassette exchange locus, human H9 ES cells with regulated Myf5 expression were generated. These were differentiated via EBs into mesenchymal cells which underwent efficient myogenesis after Myf5 induction. In this regard, Myf5 might be a better alternative to MyoD induction to avoid cell cycle arrest.

## Derivation of Myogenic Mesenchymal Cells from Human ES/iPS Cells

Recently Awaya et al. [27] reported a method for the differentiation of myogenic mesenchymal cells from human ES/iPS cells, which is somewhat similar to the strategy reported for the murine counterparts by the same group [28, 29]. Human ES/iPS cells were grown initially as embryoid bodies and then as monolayers in the presence of muscle induction medium. In this condition, clusters of Pax3^+^ or Pax7^+^ cells randomly emerged after 3 weeks, which were subsequently enriched for myogenic cells by sub-culturing on collagen-I coated plates. The peak of expression for myogenic genes was reached after 50 days in culture, at which point cells were able to terminally differentiate into multinucleated myotubes. Transplantation of these cells into immunodeficient non-dystrophic mice resulted in engraftment, as shown by the detection of human laminin-α2. Myogenic cells were able to respond to muscle re-injury and seed the satellite cell compartment to some degree. In both cases, additional transplantation studies in mouse models of MD are warranted. An advantage of this method is the fact that it does not require genetic modification to induce myogenesis. Limitations include the low efficiency of myogenic induction and the adhesion-based purification, which requires long-term *in vitro* culture.

## Future Directions

iPS technology provides a new doorway for cell-based therapies. However, there are major safety concerns associated with iPS cells, which need to be overcome before they can be seriously considered for clinical applications [30,31]. In the case of skeletal muscle regeneration, obstacles include the development of i) an efficient and safe (integration-free) myogenic induction/purification protocol, ii) safe gene correction strategies, and iii) efficient cell delivery approaches.

Because *in vitro* differentiation of pluripotent stem cells into the skeletal muscle lineage is very inefficient, most studies rely on the over-expression of genes that are critical on the regulation of the myogenic program (Pax7, Myf5 and MyoD). These approaches, though effective for differentiation, carry the potential of insertional mutagenesis since these genes are commonly delivered using lentiviral vectors.

Another point to be considered is the need for cell purification of the target cells to be transplanted since the presence of residual undifferentiated cells in these cell preparations can lead to undesired tumor formation [31]. Therefore, approaches need to be optimized to guarantee an efficient and safe method for the myogenic induction of human iPS cells in order to be considered for future applications.

Likewise, a site specific gene correction approach would be desirable for MD patient-specific iPS cells, as this technology would avoid the risks associated with random integration. Strategies involving nuclease mediated homologous recombination gene correction [17–19,32–34], are ideal to safely genetically edit and correct mutations in a site specific manner.

Finally, since the skeletal muscle is the largest organ of the human body, and in most of the MDs, multiple muscle groups are involved, local intramuscular cell injection is not a practical and feasible option [35–37]. Derivation of the appropriate myogenic population from human iPS cells endowed with efficient and selective skeletal muscle homing ability following systemic delivery is another major hurdle to overcome.

As a proof of principle, the studies reviewed above have highlighted the therapeutic potential of human iPS cells in muscular dystrophies. Overcoming the safety hurdles associated with myogenic induction and gene correction of human iPS cells will allow researchers to find practical methods for the generation of safe and clinical grade cell preparations for future therapeutic application.
